# Geocoding police collision report data from California: a comprehensive approach

**DOI:** 10.1186/1476-072X-8-72

**Published:** 2009-12-29

**Authors:** John M Bigham, Thomas M Rice, Swati Pande, Junhak Lee, Shin Hyoung Park, Nicolas Gutierrez, David R Ragland

**Affiliations:** 1 Safe Transportation Research & Education Center, University of California, Berkeley, 2614 Dwight Way #7374, Berkeley, CA 94720-7374, USA; 2 Department of Environmental Health Sciences, University of California at Berkeley, Berkeley, CA, USA; 3 Department of Epidemiology, University of California at Berkeley, Berkeley, CA, USA

## Abstract

**Background:**

Collision geocoding is the process of assigning geographic descriptors, usually latitude and longitude coordinates, to a traffic collision record. On California police reports, relative collision location is recorded using a highway postmile marker or a street intersection. The objective of this study was to create a geocoded database of all police-reported, fatal and severe injury collisions in the California Statewide Integrated Traffic Records System (SWITRS) for years 1997-2006 for use by public agencies.

**Results:**

Geocoding was completed with a multi-step process. First, pre-processing was performed using a scripting language to clean and standardize street name information. A state highway network with postmile values was then created using a custom tool written in Visual Basic for Applications (VBA) in ArcGIS software. Custom VBA functionality was also used to incorporate the offset direction and distance. Intersection and address geocoding was performed using ArcGIS, StreetMap Pro 2003 digital street network, and Google Earth Pro. A total of 142,007 fatal and severe injury collisions were identified in SWITRS. The geocoding match rate was 99.8% for postmile-coded collisions and 86% for intersection-coded collisions. The overall match rate was 91%.

**Conclusions:**

The availability of geocoded collision data will be beneficial to clinicians, researchers, policymakers, and practitioners in the fields of traffic safety and public health. Potential uses of the data include studies of collision clustering on the highway system, examinations of the associations between collision occurrence and a variety of variables on environmental and social characteristics, including housing and personal demographics, alcohol outlets, schools, and parks. The ability to build maps may be useful in research planning and conduct and in the delivery of information to both technical and non-technical audiences.

## Introduction

Each of the 50 US States maintains an electronic police collision report database. In California, the California Highway Patrol (CHP) enters data from CHP-generated reports, as well as those from local law enforcement agencies, into the Statewide Integrated Traffic Records System (SWITRS) [1]. Local police departments are required by law to forward copies of fatal or injury collision reports to CHP. Each year data from approximately 4,000 fatal collisions and 11,000 severe injury collisions are added to the system.

Collision geocoding is the process of assigning geographic descriptors, usually latitude and longitude coordinates, to a traffic collision record. On California police reports, relative collision location is recorded using a highway postmile marker (e.g., on Route 1 one-tenth mile south of postmile marker 158) or a street intersection (e.g., on A Street 75 feet west of B Street). Geocoded collision records can be mapped for visualization or can be linked to a variety of geographical data to provide more informative maps or for use in spatial statistical analyses. These data often include information on demographics or housing characteristics from the US Census Bureau, location of schools or parks, pedestrian and bicycle facilities, or other environmental or social descriptors.

The process of geocoding large collision databases requires significant technical, software, and data resources. Because of these needs, no statewide effort has been made to geocode SWITRS collision data. Efforts have been constrained to local jurisdictions and these jurisdictions generally contract work through third parties. Results of these geocoding efforts have been variable.

Other US states have developed comprehensive crash mapping and analysis systems that incorporate geocoded data as one aspect of the system. The Rutgers Center for Advanced Infrastructure and Transportation completed a statewide geocoding effort for the New Jersey Department of Transportation and built a crash analysis tool for New Jersey departments of public works [2]. The Planning Section of the Connecticut DOT geocoded the crash records for use in the statewide system developed for the Crash Outcome Data Evaluation Systems (CODES) national initiative [3]. However, published reports of these systems focus on the applications and their functionality rather than the geocoding methods.

The objective of this study was to create a geocoded database of all police-reported, fatal and severe injury collisions in California for years 1997-2006 for use by governmental agencies and injury prevention professionals.

## Methods

### Overview

The task of geocoding a SWITRS record involves translating the location information on the collision report to geographic coordinates. Records for state highway collisions have numerical valueS that correspond to the postmile system used on the California state highway system. Local road collisions are coded with the Primary and Secondary street names of the nearest intersection and the collision's direction and distance from the intersection. In addition, postal addresses are occasionally used. Table 1 shows the 5 coding scenarios that are used for the vast majority of SWITRS collisions.

**Table 1:** Types of location coding, fatal and severe injury collisions, California Statewide Integrated Traffic Records System, 2004

Coding Type	No.	%	Primary^a^	Secondary^b^	Intersection	Direction	Distance (ft.)	Side of HW	Postmile
Address	63	0.4	MAIN ST	MAIN ST 2832	-	-	-	-	-
Intersection without Offset	3,164	21.2	MAIN ST	1ST AVE	YES	-	-	-	-
Intersection with Offset	6,143	41.1	MAIN ST	1ST AVE	NO	EAST	50	-	-
Fixed Object	39	0.3	MAIN ST	LGT POLE 193	-	-	-	-	-
State Route with Postmile	5,524	37.0	ROUTE 5	-	Postmile	-	-	SOUTHBOUND	33.567
Total	14,933	100							

^a ^Primary street name

^b ^Secondary street name

^c ^Highway

The geocoding was completed with a multi-step process. First, pre-processing was performed using a scripting language to clean and standardize street name information. A state highway network with postmile values was then built from the StreetMap Pro 2003 digital street network using a custom tool written in Visual Basic for Applications (VBA) in ArcGIS 9.2 software. ArcGIS VBA provides an integrated programming environment to build tools that complement the standard ArcGIS software. Intersection and address geocoding was then performed using ArcGIS and custom tools to incorporate the offset direction and distance. Finally, additional collisions were geocoded using Google Earth Pro software. The end result of the process was the addition of latitude and longitude coordinate values to the original SWITRS data.

### Data sources

142,007 fatal and severe injury collisions on public roadways in California in 1997-2006 were obtained from the Statewide Integrated Traffic Records System (SWITRS). The fatal and severe injury collision data are used extensively by law enforcement, researchers, and injury prevention practitioners to monitor collision rates, identify hazardous locations, and develop and evaluate traffic safety programs.

Postmile locations of major intersections, entrance ramps, and exit ramps for all state highways were obtained from the California Department of Transportation's (Caltrans) Traffic Accident Surveillance and Analysis System (TASAS).

Geographic information on California highways and local roads was obtained from StreetMap Pro 2003. StreetMap Pro is a TeleAtlas-based street network that is freely available to ArcGIS software license holders and is thus a widely used street network.

A 2008 TeleAtlas-based street network was accessed through Google Earth Pro. Google Earth Pro allows a limited number of records to be imported and geocoded.

### Pre-processing

We used Perl, a text-oriented programming language, to process the collision location information prior to implementing the geocoding procedures. The pre-processing tasks involved the parsing of location information text and either (1) the modification of text or (2) the flagging of records to undergo alternate geocoding strategies. SWITRS has Primary and Secondary street name variables to code the collision location. The level of standardization is fairly high in terms of prefix and suffix abbreviations (e.g., S for 'south', RD for 'Road'). We used Perl scripts to check and improve the standardization. There was also considerable variation in the abbreviation of street names (e.g., MLK BLVD or MLK JR for 'Martin Luther King Jr Blvd'). Such records were also modified to better match the naming convention of the digital street network.

By reviewing samples of collisions that did not geocode during preliminary attempts, several text character issues were discovered. The presence of a hyphen in a street name often prevented a correct geocoding match (e.g., AVE J-5 did not geocode, whereas AVE J5 did geocode. Thus hyphens were removed from certain street names. Also, occasional erroneous symbols or characters (e.g., *.STATE ST) preceded an otherwise correct street name. These characters were removed.

Next, we flagged records for subsetting prior to geocoding. In SWITRS, the Primary street field records the street the collision occurred on, while the Secondary street field is usually the nearest crossing street. However, the California Collision Investigation Manual [4] specifies that 'identifiable landmarks' can also be entered into the Secondary street field as reference points. The landmarks can include non-standard roadways (e.g., parking lot entrances) or fixed objects (e.g., fire hydrants). Because non-standard roadways and fixed objects are not included in the digital street network, these collisions were marked as known errors and were not geocoded. Postal addresses (number and street) are also permitted in the Secondary street field. Because collisions marked with a postal address are geocodable, they were flagged to undergo a separate process.

### Postmile geocoding

In California, each state highway follows the postmile system to reference locations along the highway. The postmiles on most highways begin at 0 in the southern or western boundary of the county and increase until reaching another county boundary. Over time, highways are realigned and new postmile values are added to the beginning and end of the changed segment. For this reason, distances between postmile values may not necessarily represent the true distance in some portions of the roadway. Police officers investigating collisions on state highways record location information using intersections or posted postmiles. Reports are reviewed by Caltrans and assigned a postmile value.

Commercial digital street networks do not include postmile designations, and thus we created a state highway network with postmile values. Caltrans maintains its own linear-referenced state highway data, but in a sample of the data we determined that the network had numerous inconsistencies and thus chose to create a new network. The geocoding process for these collisions included (1) creating a base state highway network, (2) adding postmile values to the network with a custom ArcGIS tool interface, (3) checking for postmile value errors, and (4) geocoding collisions through linear referencing.

### Building a base state highway network

**Figure 1:**
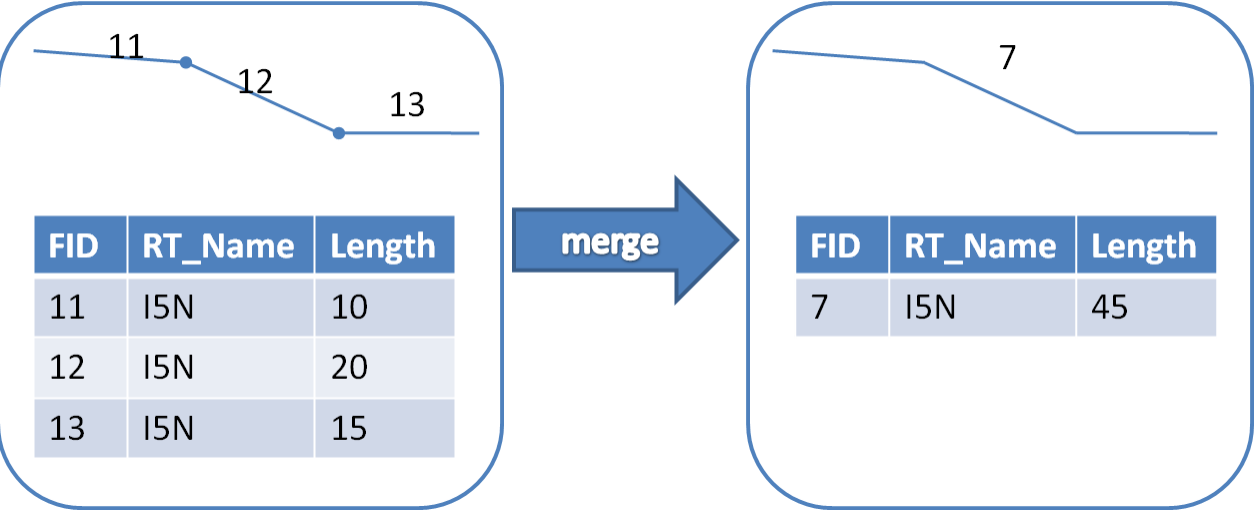
**Customization of street network included the merging of numerous short segments into one long segment**. FID = Segment ID. RT_Name = Highway route name.

The initial step in geocoding postmile-coded collisions was the development of a continuous state highway street network. Each state highway in StreetMap Pro consists of a sequence of short segments that were joined into one segment for each county in order to use ArcGIS's linear referencing functionality (Figure 1). The line features that share the same route number and direction were merged to create a single segment. This was a semi-automated process that required frequent manual intervention to deal with a range of exceptions; direction changes, two segments merging into a single segment, and naming changes. Realigned sections of the state highways also required duplicate segments to be created with overlapping postmile values. These realignments can account for changes in the roadway structure over the ten year time period of the analysis. Finally, a map layer of all counties in California was overlaid on top of the state highways layer to create unique route identifiers for each county. The identifier contains the route number, direction and county, e.g. '80E-ALAMEDA'.

### Adding postmile values to the network with a custom ArcGIS tool interface

**Figure 2:**
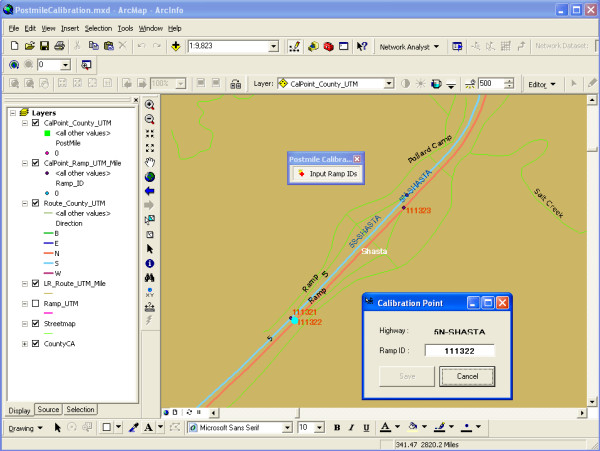
**Custom ArcGIS interface for entering postmile information**.

After building the state highway network, postmile values were added to reference points along the highways. Reference points were automatically generated where exit/entrance ramp segments intersected the state highways. Other reference points had to be manually added for county boundaries and some intersections. These reference points formed the foundation for adding the postmile values. A custom ArcGIS tool and interface were developed (Figure 2). The tool allowed users to select a reference point, open a dialog interface, and enter an ID value for a feature (e.g., an entrance ramp). The tool then saved the ID and the postmile value to the state highway network.

The postmile values added for the reference points on the street network were visually matched to the descriptive reference in the TASAS system. 15,969 postmile markers were added in total. The frequency and distance between postmile reference points varied by individual highways. Typically, interstate highways had shorter distances between reference points than small state highways, particularly those in rural or mountainous regions. To identify egregious errors in postmile values, we verified that values were progressively larger along the length of the route in each county. Each exception to this test was manually examined to verify or correct the postmile value.

### Geocoding collisions through linear referencing

Geocoding of state highway collisions was then undertaken using the linear referencing functions of ArcGIS. Linear referencing is a method of determining geographic locations using relative positions along a linear feature. If location values are known for points A and B, the value for any point between them can be determined. This calibration process was performed for the length of each state highway within each county. Postmile-coded collisions were then geocoded using the ArcGIS linear referencing tools to match the collisions to the calibrated highway network.

### Intersection & address geocoding

ArcGIS software was used to geocode intersection- and postal address-coded collisions. Collisions that did not successfully geocode were then processed with Google Earth Pro.

An address locator file was created to allow the matching of locational text to locations on the street network. The locator file used an algorithm to assign a match score to each collision record. The match score ranged from 1 to 100 and was determined by weights for each address component (i.e., street name, city, prefix, or suffix). Tests on samples of data indicated that high weights for street name and city and low weights for street type, prefix, and suffix produced the best results. Collisions that occur in unincorporated areas do not have city information in SWITRS. We treated all unincorporated areas within any one county as a zone. Because StreetMap Pro does not contain county information, we overlaid a county map layer on the street network and assigned the appropriate county to each street segment. A match score threshold was set at 65 and all geocoded records with a match score above 65, including 'ties,' were retained. The match score threshold of 65 was chosen after manually reviewing score ranges for errors. Although a match score of 65 may not be a typical threshold value, the customization of the address component weights impacted the level for acceptable scores. Records with scores from 65 to 69 (93 records from 2004) had a 6% error rate, while scores from 60 to 64 (45 records from 2004) had an 18% error rate. A score of 65 was deemed an acceptable compromise between the number of matches and match quality.

Ties could generally result from two main factors. First, major roadways are represented as dual line roads in StreetMap Pro for routing purposes. Therefore, a single intersection may have 4 points that have the same score and any of the points would be valid for the geocoding match. Second, streets occasionally intersect with another street in more than one location. It is impossible to distinguish which intersection is the actual intersection intended in the SWITRS report and therefore the intersection with the highest match score was kept.

The remaining records received low match scores for a variety of reasons: the intersection did not exist, the entry contained egregious misspellings, or the street network did not cover new or renamed streets. We explored the use of two products by Google for further geocoding--Google Maps API and Google Earth Pro. Google Earth Pro was chosen because its license allowed for batch geocoding requests for internal applications, whereas the Google Maps API licensing is restrictive for internal applications.

Google Earth Pro allows users to geocode address lists using Google's matching algorithm and a current TeleAtlas-based street network. However, Google Earth Pro does not permit a high of level of customization and cannot geocode large numbers of records. Tests showed that a high proportion of Google Earth Pro false positive matches are egregiously false. A manual review of geocoded collisions identified intersection names that were incorrectly placed. Three steps were required to maximize the Google Earth geocoding performance: (1) exclusion of intersections that were commonly placed incorrectly, (2) verification of county location, (3) adjustment of the coordinate locations to match StreetMap Pro.

Google Earth Pro often placed collisions in the wrong county when it could not locate the street information in the correct county. To identify these errors the matched data were imported into ArcGIS and the county information was verified with an overlaid county map layer. Finally, because collisions geocoded in Google Earth Pro do not exactly align with StreetMap Pro, slight adjustments were required. A process was created to snap all points within a 20 meter distance to the nearest intersection. Matches that were made on newer roads that did not exist in StreetMap Pro could not be snapped and were left unchanged. All geocoded data were then appended into a complete dataset in preparation for the calculation of offset distances where needed.

### Offset Calculation

Because 65% of intersection-based collisions in SWITRS occurred at some distance from the intersection (i.e., not at the intersection itself), geocoding and subsequent mapping efforts can be greatly improved by incorporating the offset information. The ArcGIS linear referencing tools used for the postmile geocoding can also be programmed to adjust points for each offset along the street network. We used a method similar to that of Steiner et al. [5]. First, we built a linear referencing system for all street features and then placed collisions with offset values on the features.

### Building the linear referencing system for offset calculation

The linear referencing functionality provided by ArcGIS is designed for simple linear features and cannot be directly applied to complex street networks such as StreetMap Pro. Custom programming was used to give each linear feature in the street network a unique identifier. All street segments were separated at county boundaries. Segments within the same county that were spatially connected and shared the same street name were then merged into a single feature. The South- and West-most corner of the feature was defined as the starting point at 0 distance and the distance accumulated along the entire length of the feature. The direction of the feature was determined as the greater of the vertical and horizontal distances. For example, if the vertical distance exceeded the horizontal distance, the direction was assigned as South-to-North.

Extra code was required to handle multi-lane streets that are represented as dual parallel lines travelling in opposite directions. Conceptually both lines should be considered to be the same street, but each direction had to be treated as a distinct linear feature.

### Locating collisions with offset

After creating the linear referencing system, the offset direction and distance were calculated. First, all linear features connected to the intersection point were chosen as candidate lines for the offset. Next, the features with a name that matched the Primary collision street name were selected. A single linear feature was subsequently identified by matching the direction of the offset to the direction of the linear feature in the network. Finally, the collision location was adjusted for the offset by traversing the proper distance along the linear feature.

## Results

### Geocoding Match Results

A collision geocoding attempt was considered a match if (1) an intersection- or address-coded record scored a match score of 65 or higher based on the custom ArcGIS weights; (2) an intersection- or address-coded record scored a 'tie' match score of 65 or higher; (3) an intersection-coded record was geocoded by Google Earth Pro; (4) an intersection-coded record geocoded in ArcGIS or Google Earth Pro required an offset adjustment; or (5) a postmile-coded record presented no error during linear referencing.

**Figure 3:**
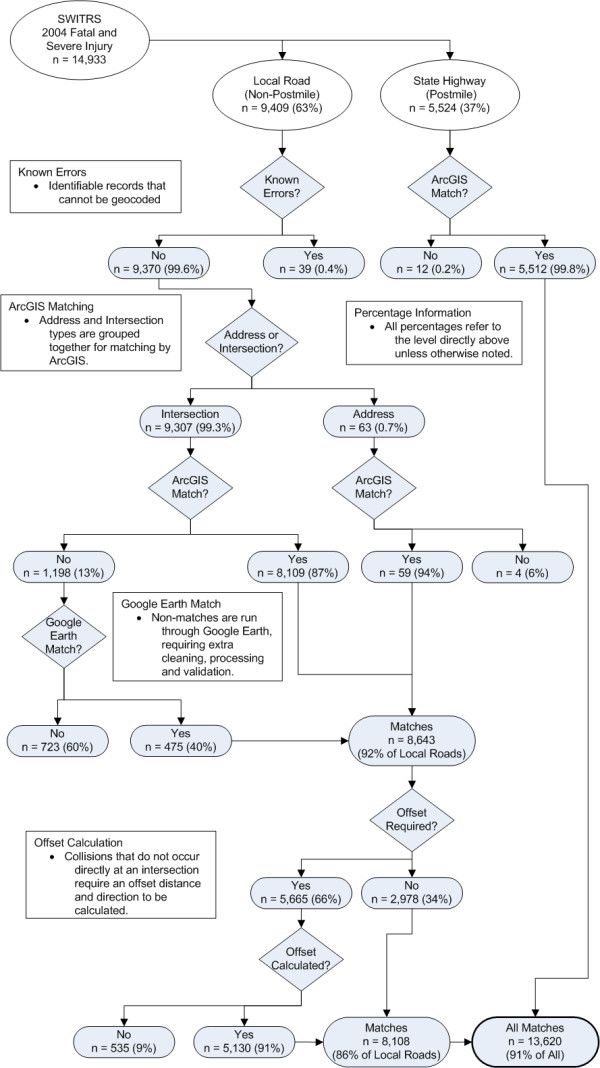
**Geocoding flow diagram, California Statewide Integrated Traffic Records**.

A total of 142,007 fatal and severe injury collisions were identified in SWITRS for years 1997-2006. Figure 3 shows the results of each component of the geocoding process for one year of data. Results for all years were similar. Of the 14,933 fatal and severe injury collision records obtained from SWITRS for 2004, 9,409 (63%) were local road collisions coded by intersection street names and 5,524 (37%) were state highways geocoded by postmile value. Overall, 13,620 (91%) were successfully geocoded.

### Intersection Geocoding

Among the 9,409 intersection-coded collisions, we identified 39 that had location information with references to utility poles and other fixed objects on which we had no information. These collisions could not be geocoded. An additional 63 collisions contained postal addresses. Of these, 59 were geocoded. 8,109 of the remaining 9,307 intersection-coded local road records (87%) were geocoded to the nearest intersection using ArcGIS. 2,727 of these geocoded collisions (34%) were ties. Of the 1,198 non-matching records, 475 (40%) were geocoded using Google Earth Pro. Of all intersection-coded collisions, a total of 8,643 (92%) were geocoded.

### Intersection Offset

Of these 8,643 geocoded collisions, 5,665 (66%) contained offset information. We adjusted for the offset direction and distance for 5,130 (91%) of these but were unable to do so for 535 (9%) records. For example, many records had an offset distance that would place the collision beyond the end of the street.

### Postmile Collisions

There were 5,524 postmile-coded State Highway collisions in the 2004 data file. Of these, 5,512 (99.8%) were geocoded. Collisions that could not be geocoded included those with egregious postmile errors, incorrect route numbers (nonexistent within county), or those that occurred on newly constructed highways not yet included in the street network.

### Positional Accuracy

After completing the geocoding process, an estimate of the positional accuracy was calculated using a random sample of 500 local road collisions. Positional accuracy was measured relative to the Google Earth Pro street network. The sample of local road collision records was manually reviewed in Google Earth Pro to assess accuracy. If the record contained offset information, we used the measuring tool to estimate offset distance. Due to the inexact nature of a manual measurement, any measurements within 50 feet of the recorded distance were considered correct. Of the 500 collisions, 489 (97.8%) had correct primary and secondary roads and the offset distance and direction appeared to be correct.

## Discussion

We were able to geocode 91% of 142,007 California fatal and severe injury collisions identified in SWITRS for years 1997 to 2006. The effort resulted in a statewide database of geocoded collision data that should prove useful for injury prevention research and practice. A second primary outcome was the creation of a postmile-based, digital street network for the California state highway system to facilitate the use of linear referencing methods for geocoding highway collisions or other events or objects. The use of this street network in the current project allowed for a geocoding success rate of 99.8% for state highway collisions, compared with 86% of collisions geocoded by intersection.

The geocoding of postmile-coded collisions is dependent on the quality of the postmile information obtained from SWITRS. There are numerous opportunities for error in the collection and processing of postmile information. First, police officers may incorrectly code the collision location on the collision report. Second, Caltrans may provide an incorrect postmile value for the information on the collision report. We had no way to identify these errors. Third, the postmile geocoding is based on the linear referencing system of the StreetMap Pro network. The network's postmile values or linear referencing process may have introduced errors. Other street networks lack postmile information and thus cannot be used to examine these potential errors. We were able to identify only egregious errors in the state highway postmile system that caused multiple collisions to be placed in implausible locations. Aside from these cases, we assumed all postmile values to be correct. We recognize that this assumption was not always met.

**Figure 4:**
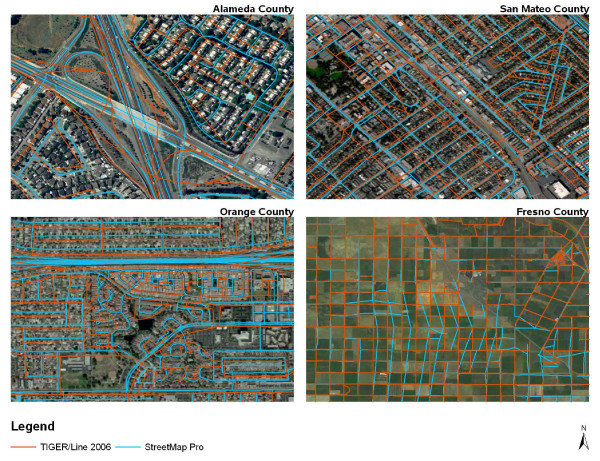
**Visual comparison of StreetMap Pro 2003 and TIGER/Line 2006 street network**.

StreetMap Pro was chosen for this project because of its high level of accuracy in urban areas, its accurate representation of highway interchanges, and aspects of its license terms. StreetMap Pro is freely licensed with ArcGIS software and is widely used by the GIS community. Thus many of the processes developed in this project will be extensible to other geocoding efforts. StreetMap Pro and the TIGER/Line street network from the US Census Bureau were selected for consideration. Other commercial networks were not considered due to their restrictive license terms or prohibitive costs. In a recent paper [6], Frizelle et al. compared StreetMap Pro to the TIGER/Line 2007 street network in a rural setting and found StreetMap Pro to be of low quality. To compare street network quality in California, we compared 10 samples each of urban and rural areas across the state using StreetMap Pro and TIGER/Line 2006. We found StreetMap Pro to be superior in urban areas, while TIGER/Line 2006 was generally more accurate in rural areas. Figure 4 shows example locations in four California counties with the street networks overlaid on current imagery from Microsoft Bing Maps. StreetMap Pro more closely matched the imagery in the urban areas of Alameda, San Mateo, and Orange counties, compared with TIGER/Line 2006. The Alameda County map especially illustrates the marked deficiency of TIGER/Line 2006 at large highway interchanges. High quality interchanges were essential for development of the postmile linear referencing system. In Fresno County, TIGER/Line 2006 is relatively accurate while gross deviations are evident in StreetMap Pro. TIGER/Line 2006 also had the ability to incorporate newly added roads that were not present in StreetMap Pro. Despite these potential benefits of TIGER/LINE 2006, StreetMap Pro represented the best choice due to the quality of the urban areas and highway interchanges.

The results of this project compared favorably to the other published reports (Table 2). We matched 86% of local roads and tests showed that 98% were correct. Our postmile-based state highway geocoding matched 99.8% of collisions without any recognizable placement errors. Although the methodologies in the reports in Table 2 were subsequently applied to larger crash databases in their respective areas, the datasets used for the published results were all restricted by geography, road type, or collision type. Our results included all fatal and severe injury collisions on all of California's public roadways. Also, only Dutta et al. provided specific results of an accuracy assessment of local road collision geocoding. They reported a 2.5% error rate, which is consistent with our rate.

**Table 2:** Geocoding match results, selected studies

Author	Collisions	Road Types	Crash Types	% Geocoded	% Incorrect	Scale	Location
Dutta et al (4)	4,351	Local Roads	All	78.5	2.5	State	Wisconsin
Steiner et al (5)	1,756	All	Pedestrian	84.6*	n/a	County	Miami Dade Co, FL
Zhan et al (6)	35,531	Highway	All	97.9	n/a	County	Palm Beach Co, FL
Zhan et al (7)	59,247	Highway	All	95.6	n/a	County	Broward Co, FL

*10.3% more were manually geocoded

The collision match rates varied for urban vs. rural areas. The collision match rate was 75% for unincorporated areas, compared with 90% for incorporated cities. The low geocoding rate in unincorporated areas may result from the difficulty in recording collision location in these rural settings or lower quality street networks. Match rates did not vary by collision severity--91% of fatal collisions, compared with 90% of severe injury collisions. We do anticipate lower match rates in our follow-up study of minor injury collision geocoding in California, because the extent and quality of police investigations is generally thought to be lower for collisions resulting in less severe injuries.

An on-going follow-up study will enhance our geocoding process by improving the state highway street network, performing manual geocoding of unmatched data from selected jurisdictions, improving the pre-processing of police collision report location information, and streamlining the processes into one user interface. We will apply this improved geocoding process to all California minor injury collisions (180,000 per year) in addition to the fatal and severe injury collisions used in this work.

GPS technology is available to allow law enforcement agencies to capture geographic coordinates during traffic collision investigations. It appears unlikely that the universal use of this technology in California patrol vehicles will be achieved in the near future. Preliminary results of an ongoing survey of California police departments indicate that only 7% of departments currently equip all patrol vehicles with GPS units and 22% equip some but not all vehicles with them. Periodic geocoding processes like the one reported here will continue to be necessary even in jurisdictions that adopt universal GPS use. Sarasua et al[7] evaluated South Carolina's effort to collect GPS from all collision-reporting patrol vehicles in that state and found that 80% of collision reports had adequate-quality geographic data. It appears that collision geocoding needs will be greatly reduced but not eliminated by GPS technology.

Our study can provide a framework for geocoding efforts in other countries. It appears that most developed countries use one or more of address-, intersection-, parcel-, and highway marker-based location coding during collision reporting. Our methods could be adapted to geocode collisions in a jurisdiction that uses any of these coding schemes. For example, our code could be modified to offset collisions from postal address reference points. Intersections are of course a more common reference point used in collision reporting, and our offset code could easily be used with locally available street networks. For the highways or expressways, the approach and custom tools for developing a marker-based linear referencing system could be applied in most cases. For example, the methodology from this project has been used by one of the authors to build a postmile-based linear referencing system for Korean expressways.

Geocoded collision data are often used by researchers and practitioners in the fields of traffic safety and public health. For example, transportation engineers have investigated collision clusters along state highways and their relation to carpool lanes, road construction projects, wet road surface, or other factors. Public health researchers have examined alcohol-involved collisions with respect to retail alcohol outlet density [8] and neighborhood demographics [9]. Child pedestrian injuries have been correlated with school locations [10]. Many geocoding efforts involve full or partial manual geocoding and are thus generally very limited geographically or temporally. Regional or national databases of geocoded collision data will facilitate the incorporation of geographic analyses into traffic safety and injury prevention programs. The geographic information associated with traffic collision records, regardless of source, has the potential to inform and support traffic safety and injury prevention programs and research. The use of GIS methods to study traffic collisions is likely to improve research and program planning, to inform policy, and to contribute to overall improvements in public health.

## Competing interests

The authors declare that they have no competing interests.

## Authors' contributions

JB conceptualized the study and methods, developed the intersection geocoding process, oversaw all GIS processes, and drafted the manuscript. TR conceptualized the study and methods, obtained funding, and revised the manuscript. SP developed pre-processing methods and scripts. JL developed methods and scripts for customizing the road network. SHP built the base state highway network and developed the custom postmile input tools. NG developed pre-processing methods and scripts. DR developed methods and performed exploratory data analysis. All authors read and approved the final manuscript.
